# Amplification of select autonomous HERV loci and surrounding host gene transcription in monocytes from patients with post-acute sequelae of COVID-19

**DOI:** 10.3389/fimmu.2025.1621657

**Published:** 2025-06-26

**Authors:** Hyunmin Koo, Casey D. Morrow

**Affiliations:** ^1^ Department of Genetics, Hugh Kaul Precision Medicine Institute, Heersink School of Medicine Immunology Institute, University of Alabama at Birmingham, Birmingham, AL, United States; ^2^ Department of Cell, Developmental and Integrative Biology, Hugh Kaul Precision Medicine Institute, Heersink School of Medicine Immunology Institute, University of Alabama at Birmingham, Birmingham, AL, United States

**Keywords:** post-acute COVID sequelae, monocytes, epigenetic remodeling, ScRNA-seq, human endogenous retrovirus (HERV)

## Abstract

**Background:**

The human genome contains approximately 3,200 near full-length autonomous human endogenous retroviral (HERV) genomes distributed across the 23 chromosomes. These autonomous HERV proviral genomes include long terminal repeats (LTRs) capable of promoting RNA transcription. In quiescent cells, most HERV loci remain transcriptionally silent. However, environmental changes, such as epigenetic remodeling of chromatin, can activate these silenced loci.

**Methods:**

To study HERV reactivation, we previously analyzed autonomous HERV expression patterns in monocytes isolated from peripheral blood mononuclear cells (PBMCs) identified in single-cell RNA sequencing (scRNA-seq) databases using the Azimuth application. We developed a Window-based HERV Alignment (WHA) method, which analyzes aligned DNA sequences using sequential, non-overlapping windows of defined lengths. Samples were scored as positive (>= 9 good/usable windows) or negative (<= 8 good/usable windows).

**Results:**

Using WHA, we established a control set from 31 normal individuals, with fewer than 8 windows at selected HERV loci. We analyzed scRNA-seq data from three studies of hospitalized COVID-19 patients and found distinct HERV expression patterns in monocytes. Unique patterns were also found in patients with influenza, Dengue virus, or sepsis. We next examined HERV expression at early (<7 days) and late (>14 days) timepoints post COVID-19 recovery and detected HERV loci in both groups. Analyzing 12 patients with post-acute sequelae of COVID-19 (PASC), we identified three HERV loci expressed in all patients. Some loci showed amplified numbers of good/usable windows, indicating longer transcripts and greater sequence depth. The most amplified locus was located within an intron of JAKMIP2, which, along with neighboring host genes, also showed increased transcription.

**Conclusion:**

Previous studies have shown that viral infections, including COVID-19, influenza, and Dengue virus, as well as sepsis, can induce innate immune memory in monocytes through epigenetic remodeling of hematopoietic stem and myeloid precursor cells. The identification of co-amplified HERV loci and neighboring host gene transcripts in monocytes from PASC patients suggests expansion of epigenetically remodeled myeloid progenitors. The identification of these HERV-host gene patterns provides a foundation needed to understand the clinical features of patients with PASC.

## Introduction

Studies have established the importance of innate immune cells, such as monocytes, in the response to various viral and bacterial pathogens (1–3). Viral infections, including COVID-19, influenza, and Dengue virus, as well as bacterial infections (sepsis), have been shown to alter the function of monocytes (4–13). Furthermore, there is growing recognition of the ability of these viral infections to induce innate immune memory, that include epigenetic remodeling of bone marrow-derived hematopoietic stem cells and myeloid precursor cells (14–20). These epigenetically remodeled myeloid precursors have a limited capacity for self-renewal, leading to their expansion following primary viral infection (20–23).

The human genome contains approximately 3,200 near full-length autonomous human endogenous retroviral (HERV) genomes, which are distributed across the 23 chromosomes (24, 25). These autonomous HERV proviral genomes contain long terminal repeats (LTR) capable of promoting RNA transcription (25, 26). In quiescent, normal cells, only a subset of these autonomous HERV loci is transcriptionally active, while the most of the HERV loci are silenced in the resting cell (27–29). In a previous study, using Single-cell RNA sequencing (scRNA-seq) databases we analyzed the expression patterns of the autonomous HERVs in monocytes identified in resting and *in vitro* stimulated peripheral blood mononuclear cells (PBMC) (30). We developed a method called Window-based HERV Alignment (WHA), which analyzes aligned DNA sequences using sequential, non-overlapping windows of defined nucleotide lengths. Samples are scored as either positive (9 or more good/usable windows detected) or negative (8 or fewer good/usable windows) (30) (See **Supplementary Method**). We established a control set consisting of 31 normal individuals identified from several different scRNA-seq analyses, in which the numbers of windows were fewer than 8 for selected HERV loci. Using this analysis, we found positive HERV loci expression patterns in monocytes by analyzing scRNA-seq datasets from PBMCs obtained from individuals with trauma or COVID-19, both of which are known to stimulate *in vivo* monocyte transcription. Since HERV expression is sensitive to gene silencing, our system provides a useful tool for assessing the impact of different pathological conditions that lead to the *in vivo* expression of previously silenced HERV loci in innate immune cells, such as the monocyte (31, 32).

In the current study, we used these 31 individual data sets as a pangenome control to compare the autonomous HERV loci transcriptomes in monocytes from patients with different diseases. WHA analysis revealed that the monocyte populations from acutely infected patients with COVID-19, influenza, Dengue virus, or sepsis exhibited distinct patterns of autonomous HERV loci transcriptome expression compared to the 31-pangenome controls. Additionally, using a dataset from patients with post-acute sequelae of severe acute COVID-19 (PASC) at 8 months post-acute infection, we identified a common set of HERV loci in monocytes present in all 12 patients. A subset of these HERV loci exhibited an increased number of good/usable windows (i.e., sequence length) and greater sequence depth compared to the same loci found in hospitalized patients with acute COVID-19 infections, who tested positive by antigen or RT-PCR. The most amplified HERV loci transcriptome was in the first intron of a cellular gene, JAKMIP2. Further analysis revealed the JAKMIP2, along with other nearby host genes in Chromosome 5 also had significantly increased expression. The patterns of co-amplified HERV loci with surrounding host gene transcripts in monocytes in patients with PASC are consistent with the establishment of trained innate immunity that is characterized by localized epigenetic remodeling of myeloid precursors (20, 33).

## Results

In a previous study, we described Window-based HERV Alignment (WHA), in which DNA from scRNA-seq analysis is aligned to the 3,200 autonomous HERV loci in non-overlapping windows of defined nucleotide lengths (30). Usable windows are defined as those with a read depth of 3 or greater and must have a minimum of 9 good/usable windows, corresponding to an extended (i.e., longer) expression of HERV transcripts. In contrast, those HERV loci designated as negative (8 or less windows) do not meet the necessary sequence read depth or number of good/usable windows as determined by WHA. We used the WHA to develop a pangenome control that consisted of scRNA-seq datasets from 31 normal individuals taken from multiple studies. The WHA data was then filtered to eliminate any of the 3,200 HERV loci that had at least one positive (i.e., WHA of greater than 9 windows) in the 31-pangenome samples (30) (See **Supplementary Method**).

### HERV loci expression in monocytes from COVID-19 exposed, infected with no symptoms and acutely infected hospitalized patients

**Figure 1:**
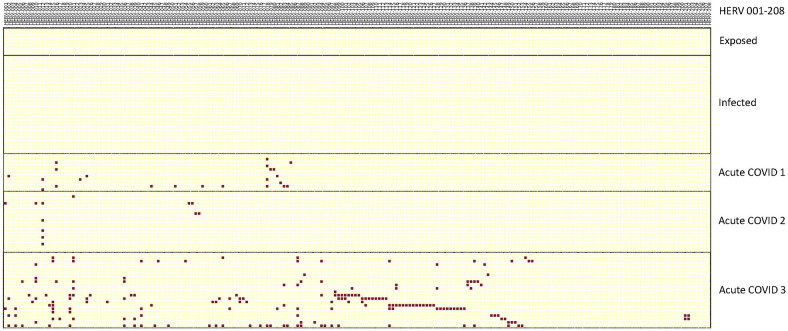
Distribution of positive HERV loci found in exposed, infected and three datasets of acutely infected COVID-19 patients. The positive HERV loci for the data sets for the exposed, infected and acutely infected patients is depicted in a vertical column. HERV loci that were accessed were depicted in a horizontal column. To identify the positive HERV loci in these datasets, we compared them with the pangenome control dataset consisting of 31 normal individuals, as previously described (30). Positive HERV loci in the PBMC datasets were identified through comparison with the normal pangenome control set. Red boxes indicate positive HERV loci, while yellow boxes indicate negative HERV loci below cut-off values for depth and windows count. Numbers of individuals per each group; 3 exposed COVID-19 individuals, 10 infected COVID-19 individuals, Acute 1: 11 patients; Acute 2: 10 patients; Acute 3: 12 patients. Note some samples contained multiple time points – see **Supplementary Table 2** for detailed information on identity of each HERV locus.

In the first analysis, we used three scRNA-seq datasets from hospitalized acute COVID-19 patients, along with an scRNA-seq dataset from individuals exposed to or infected with COVID-19 who showed no symptoms (7, 13, 34, 35). Each of these scRNA-seq datasets, along with the pangenome datasets, was first reanalyzed using *Azimuth* with the specific reference sequences used for PBMC (36, 37). We analyzed the PBMCs from the patients for monocytes and used the WHA to identify positive HERV loci. We then filtered these HERV loci against the normal individual controls for each study to identify those that were positive in the patients but negative in the controls. Using the comparison, we found no positive HERV loci in the individuals who were exposed to, or showed no COVID-19 symptoms (34) (**Figure 1**).

In contrast, we found positive HERV loci in monocytes from three independent studies of acutely infected, hospitalized COVID-19 patients. The patients in Lee et al. (herein referred to as Acute 1 group) and Unterman et al. (herein referred to as the Acute 2 group) recovered after hospitalization. In contrast, in Amrute et al. (herein referred to as the Acute 3 group), one set of patients recovered after hospitalization, while another set did not recover and died (**Supplementary Table 1**) (7, 13, 35). Analysis of autonomous HERV loci expression revealed that all three studies had different numbers of positive HERV loci. However, no individual HERV locus was found in all samples of a particular group and no HERV locus shared across all three acute datasets. Nonetheless, in several instances, we found HERV loci shared between individuals within the same dataset (**Figure 1**, **Supplementary Table 2**). Furthermore, in the Acute 3 samples set, 13 pairs of samples from patients taken on day 0 and day 7 contained the same positive HERV loci, indicating sustained expression of these specific HERV loci in these individuals. Thus, consistent with our previous study, the patterns of HERV loci expression in the three sets of hospitalized acute COVID-19 patients are individual-specific and vary in the number of positive HERV loci (30).

### Analysis of HERV loci expression in monocytes obtained from patients with different disease or vaccinated normal individuals

**Figure 2:**
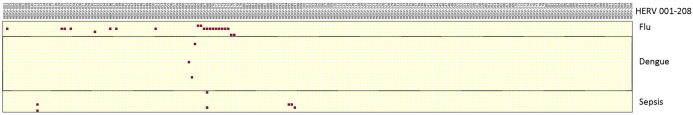
Distribution of positive HERV loci found in patients infected with influenza, dengue virus or sepsis. The positive HERV loci found in PBMCs from patients infected with influenza, Dengue virus or sepsis is depicted in a vertical column. HERV loci that were accessed were depicted in a horizontal column. Red box indicates a positive HERV loci, yellow indicates a negative HERV loci below cut-off values for depth and windows. Numbers of patients per each group; Flu: 5, Dengue virus: 15, Sepsis: 4. Note some samples contained multiple time points – see **Supplementary Table 3** for detailed information on identity of HERV loci.

**Figure 3:**
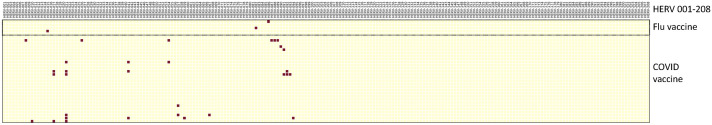
Distribution of positive HERV loci found in individuals who were administered either the influenza or COVID-19 vaccines. The positive HERV loci found in PBMCs from patients who were administered either the influenza or COVID-19 vaccine is depicted in a vertical column. HERV loci that were accessed were depicted in a horizontal column. Red box indicates a positive HERV loci, yellow indicates a negative HERV loci below cut-off values for depth and windows. Number of individuals per each group; Flu vaccine: 1, COVID vaccine 6. Note some samples contained multiple time points – see **Supplementary Table 4** for detailed information on identity of HERV loci.

We next expanded the analysis to include scRNA-seq datasets from hospitalized patients with severe influenza, acute respiratory sepsis, and Dengue virus (**Figure 2**, **Supplementary Table 3**) (6, 35, 38). We identified positive HERV loci in 4 of the 5 hospitalized patients with severe influenza, with one patient having 16 positive HERV loci. In contrast, we found positive HERV loci in only 4 of the 7 sepsis patients and 3 of the 19 patients with dengue virus. To determine whether viral infection was necessary, we analyzed a dataset of COVID-19 immunized individuals. We found HERV loci expression in individuals who had received the COVID-19 vaccine (**Figure 3**, **Supplementary Table 4**). In this case, 6 individuals were given the vaccine, and their PBMCs were analyzed using scRNA-seq (39). We found that 4 of the 6 individuals exhibited HERV loci expression at various times post-vaccination. Interestingly, two patients also had HERV loci expression at time 0, suggesting they might have had prior exposure to COVID-19. As with the acute samples, the patterns of HERV loci expression in these 4 individuals varied with respect to timing after vaccination. Thus, the results from the vaccine analysis indicate that infection *per se* is not necessary to stimulate the expression of certain HERV loci, although viral respiratory infections such as COVID-19 and influenza result in more extensive HERV loci expression.

### Analysis of HERV loci expression in patients post acute COVID-19 infection

**Figure 4:**
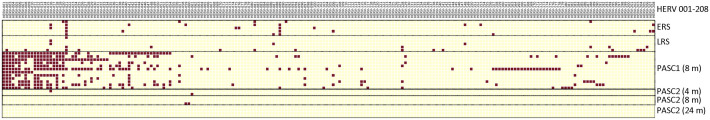
Distribution of positive HERV loci found in individuals with early or late recovery, or those identified as PASC. The positive HERV loci found in PBMCs from patients post COVID-19 infection, categorized as early, late, or identified as PASC is depicted in a vertical column. HERV loci that were accessed were depicted in a horizontal column. Red box indicates a positive HERV loci, yellow indicates a negative HERV loci below cut-off values for depth and windows. Number of patients per each group; ERS: 5, LRS: 5, PASC 1 (8m): 12, PASC 2: 2. Note some samples contained multiple time points – see **Supplementary Table 5** for detailed information on identity of HERV loci.

We next analyzed a scRNA-seq dataset from patients at early times post-acute COVID-19 infection. In the first dataset, two patient groups were analyzed for HERV loci expression: an early group that became RT-PCR negative at or before 7 days and a late group that became RT-PCR negative at 14 days or later (40) (**Figure 4**, **Supplementary Table 5**). In both groups, we found positive HERV loci with a sporadic pattern of HERV expression in both early and late infections. However, several HERV sites were common to both groups. The most frequently shared HERV locus HERV-021, which was positive in all 5 early samples and 3 of 5 late samples. The second most common, HERV-089, was found in 4 of the 5 early samples but was 0 in all of the 5 late samples (**Figure 4**, **Supplementary Table 5**).

Numerous studies have described patients with post-acute sequelae of COVID-19 (PASC), also known as long-term COVID-19 (16, 17, 41–46). We analyzed 12 samples from PASC patients taken 8 months post-acute COVID-19 as part of the larger, well-characterized Long-term Impact of Infection with Novel Coronavirus (LIINC) study (46). The patients were adults (greater than 18 years old) with greater than 2 weeks past onset of COVID-19 symptoms or, if the patients were asymptomatic, the first positive diagnostic test. During the post-acute phase, the patients had multiple symptoms associated with PASC including fatigue, shortness of breath, concentration problems, headaches and trouble sleeping that were most common through the 8 months of observation (41, 42, 46) (**Supplementary Table 1**). For our analysis, PBMCs from these 12 patients were analyzed by scRNA-seq (46). Notably, three HERV loci (HERV 001, 002, and 003) were positive in all 12 patients, while 11 of the 12 patients had positive HERV loci (HERV 011, 012, and 018). In a second analysis, we used a scRNA-seq dataset from a different study that examined PASC patients at 4 months, 8 months, and 2 years (47). In this study, most participants had their first visit between 2 and 3 months after their initial diagnosis, with follow-ups at month 4, month 8, and year 2. PASC individuals were identified based on the presence of at least one of three major symptoms (fatigue, dyspnea or chest pain) at month 4 (**Figure 4**, **Supplementary Table 5**). An additional challenge in the scRNA-seq analysis was that control samples from normal individuals in this study and PASC samples at 4 months, 8 months, and 2 years were combined at a 1:1 ratio. From our analysis, we identified a few positive HERV loci at the 4- and 8-month time points and found no patients with positive HERV loci at 24 months. Thus, despite the complication of mixing of control and experimental samples, we still detected positive HERV loci a 4 and 8 months but none at 24 months.

In summary, we found that the three acute COVID-19 sample sets (totaling 51 samples) and the 12 samples from PASC patients had the highest number of unique HERV loci sites (**Supplementary Table 6**). A comparison between acute COVID-19 and PASC samples revealed 69 unique HERV loci in the acute samples and 52 unique loci in the PASC samples. Additionally, 51 positive HERV loci were identified in both acute and PASC samples, however, no single HERV locus was found in all acute and PASC patients.

**Figure 5:**
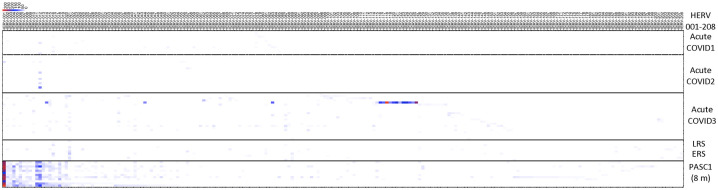
Distribution of positive windows found from analysis of samples from acute patients, early or late recovery patients and PASC patients. The number of the positive HERV loci for each locus were compiled and presented in a heatmap. The 8-month PASC patient sample set contained the greatest number of positive HERV loci. See **Supplementary Table 7** for detailed information on identity of HERV loci.

**Figure 6:**
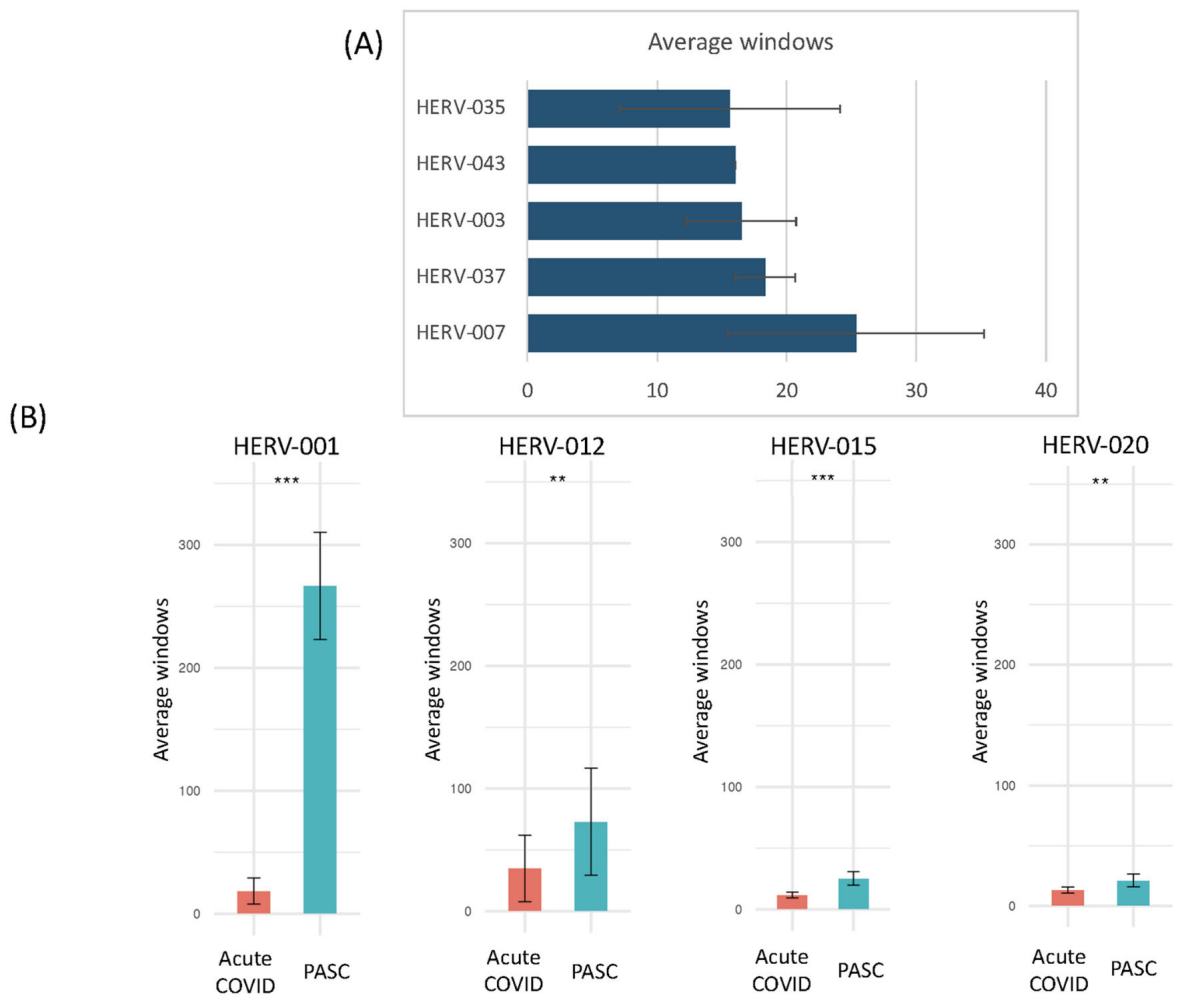
Comparison of the number of positive windows obtained from PASC patients and those found in the remaining datasets (acute, vaccinated, sepsis, influenza, Dengue virus, influenza virus and 24-month post-COVID). The number of positive windows for Individual HERV loci in the sample set from 8-month PASC patients was compared with the combined number of positives for the same loci from the remaining datasets. **(A)** Positive HERV loci identified at specific loci in the 8-month PASC samples with no positive HERV loci detected in any other datasets. The average number of good/usable windows, along with the standard deviation, observed in the PASC samples is shown in the figure. **(B)** Positive HERV loci identified at specific loci in the 8-month PASC samples compared to those detected at the same loci in the remaining datasets. The average number of good/usable windows for the top HERV loci in the PASC group was used to perform a t-test against the acute group. The number of positive windows for 1) HERV001 loci in PASC samples versus those found in the other samples 163-7 (Acute 3, Amrute et al.), TS4-A (Acute 2, Unterman et al.), 2) HERV015 loci in PASC samples versus those found in the other samples 80-0, 80-7, 154-0, 163-0, 163-7, 251-0, 251-7 (Acute 3, Amrute et al.), 3) HERV012 loci in PASC samples versus those found in the other samples S1-nCOV1, S12-nCOV6 (Acute 1, Lee et al.) and NS-1A, TP-6A, TP-7A, TP9-B, TS-4A, 4) HERV020 loci in PASC samples versus those found in the other samples 145-0, 163-0, 163-7,272-0, 272-7 (Acute 3, Amrute et al.). "**" = p-value < 0.05, "***" = p-value < 0.001.

To highlight the differences between acute, the early/late recovered (ERS/LRS), and the PASC samples, we analyzed the number of good/usable windows identified for each positive HERV locus (**Figure 5**, **Supplementary Table 7**). Overall, there was a clear difference in the distribution of HERV loci, with PASC samples exhibiting a higher number of good/usable windows compared to acute or early/late samples. The difference was most evident in HERV loci 001-017. In the acute samples, we identified one individual (251-0) with a cluster of HERV loci (HERV 114-121) showing a high number of good/usable windows, such as HERV-118 with HERV-339 good/usable windows. Interestingly, this elevated number of good/usable windows was not observed in the same individual 7 days later (251-7). To quantify these differences, we compared the number of good/usable windows in acute versus PASC samples. We identified 5 HERV loci present only in PASC patients (**Figure 6A**). The average number of good/usable windows for these 5 HERV loci ranged from 10 to 25. Additionally, we identified 6 HERV loci (HERV001, 004, 006, 012, 015, and 020) where the number of good/usable windows in PASC samples was significantly greater than in acute sample (**Figure 6B**, **Supplementary Figure 1**).

One of the most striking results was found from the analysis of HERV001, which was detected in all 12 PASC patients with good/usable window counts ranging from 172 to 332 that were all significantly different than that found in patients with acute COVID-19. In contrast, we found 5 HERV loci (HERV025, 054, 057, 097, and 106) that exhibited similar numbers of good/usable windows between acute and PASC samples, with no significant p-value observed (**Supplementary Figure 2A**). Additionally, 5 HERV loci (HERV083, 067, 087, 065, and 114) were detected only in the remaining COVID-19 patient samples and not in the PASC sample (**Supplementary Figure 2B**).

**Figure 7:**
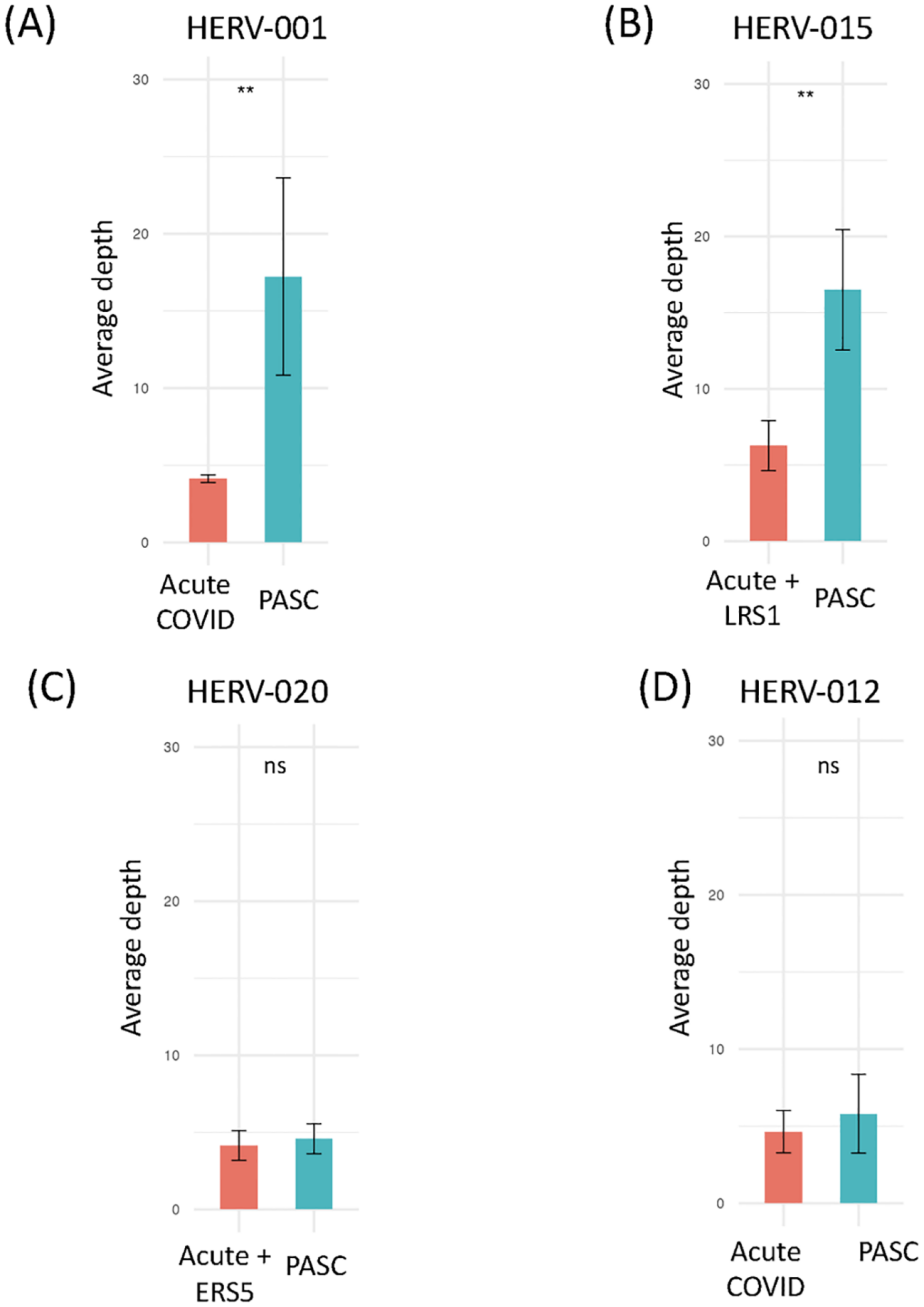
Comparison of the sequence read depths of PASC patients at the same HERV loci in the remaining datasets. Comparison of the read depths for four HERV loci between the PASC dataset and the acute, vaccinated, sepsis, influenza and Dengue virus datasets using t-test (**Supplementary Table 7**). Any statistically significant p-value (p < 0.05) are shown in the figure (‘ns’ indicates not significant). **(A)** The sequence read depth for HERV001 loci in PASC samples versus those found in the acute sample 163-7 (Acute 3, Amrute et al.) and TS4A (Acute 2, Unterman et al.). **(B)** The sequence read depth for HERV015 loci in PASC samples versus those found in samples 80-0, 80-7, 154-0, 163-0, 163-7, 251-0, 251-7 (Acute 3, Amrute et al.) and LRS1 (ERS/LRS, Wen et al.). **(C)** The sequence read depth for HERV020 loci in PASC samples versus 145-0, 163-0, 163-7,272-0, 272-7 (Acute 3, Amrute et al) and ERS5 (ERS/LRS, Wen et al.). **(D)** The sequence read depth for HERV012 loci in PASC samples versus samples S1-nCOV1, S12-nCOV6 (Acute 1, Lee et al.) and NS-1A, TP-6A, TP-7A, TP9-B, TS-4A (Acute 2, Unterman et al.). "**" = p-value < 0.001, "ns"= not significant.

We next examined the sequencing read depth of positive HERV loci samples. We found that 2 HERV loci (HERV001 and 015) had significantly higher read depth in PASC samples compared to acute COVID-19 samples (**Figure 7**). In contrast, no significant difference in read depth was observed between acute and PASC samples for HERV020 and HERV012 (**Figure 7**, **Supplementary Figure 3**). These findings suggest two distinct patterns of HERV expression when comparing acute and PASC sample sets: (1) a pattern in which both the number of good/usable windows and sequencing depth are significantly greater in PASC samples compared to acute samples, and (2) a pattern in which PASC samples have significantly more good/usable windows than acute samples but without a significant difference in sequencing read depth.

**Figure 8:**
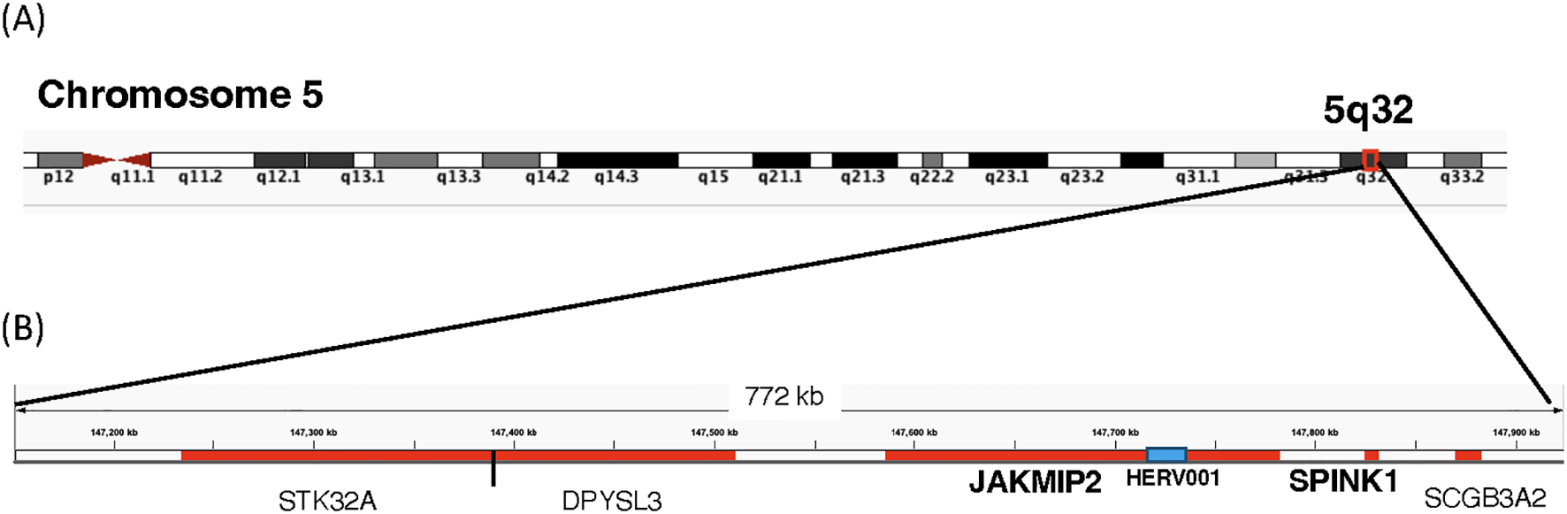
Location of Host of HERV-001 and surrounding genes on Chromosome 5. **(A)** Chromosome 5 is shown with the subregion 5q32 indicated. A red box highlights the genomic region containing HERV-001 and its neighboring host genes. **(B)** An expanded view of this 772 kb region includes the host genes JAKMIP2, DPYSL3, SPINK1, STK32A, and SCGB3A2 (highlighted in red). HERV-001, located within intron 1–2 of JAKMIP2, is marked with a blue box and annotated as HERV-001 (intron of JAKMIP2). The bolded JAKMIP2-HERV001-SPINK1 reflects the localized amplification of the expression of these genes in the monocytes from the PASC.

We next mapped the HERV loci identified in **Figures 6A, B** to the 23 chromosomes (**Supplementary Figure 4**). Overall, we did not observe clustering of the identified HERV loci; instead, they were in regions of the chromosomes that were not devoid of HERV loci. Chromosome 1 contained the highest number of HERV loci (2), while Chromosomes 2, 3, 5, 6, 7, 12, and X each contained a single HERV loci. HERV001 was mapped to chromosome 5 within an intron 1–2 of the janus kinase and microtubule interacting protein 2 (JAKMIP2) gene (**Figure 8**). In contrast, HERV015 was mapped to Chromosome 2 in an independent region that was near the Mer proto-oncogene tyrosine kinase (MERTK) which is highly expressed in macrophages, monocytes, and progenitor cells (48). HERV012 was mapped to chromosome 6, located 5’ of the solute carrier family 16-member 10 (SLC16A10), while HER020 was mapped to Chromosome X within an intron of the gene proline-rich and glia domain 1 (PRRG1) gene (**Supplementary Table 8**).

**Figure 9:**
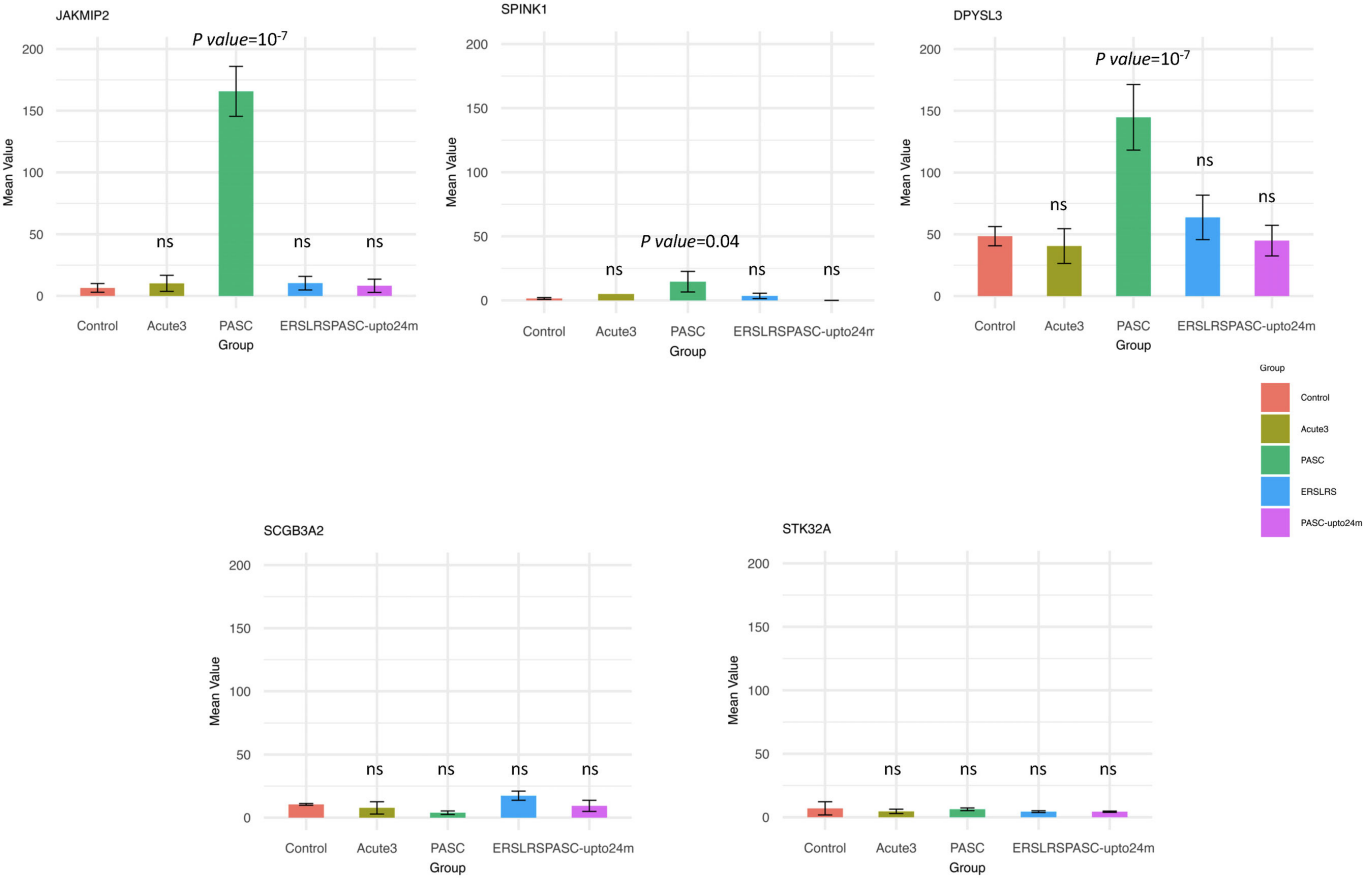
Analysis of host gene expression in control, acute, early/late, PASC 24 month and 8 month PASC. For each host gene, the number of good/usable windows observed in each patient group was compared to the Control group using ANOVA followed by Tukey’s HSD test. Any statistically significant p-value (p < 0.05) are shown in the figure (‘ns’ indicates not significant). From this analysis, we identified 3 genes (DPYSL3, JAKMIP2, and SPINK1) with significantly higher numbers of good/usable windows in the PASC group.

**Figure 10:**
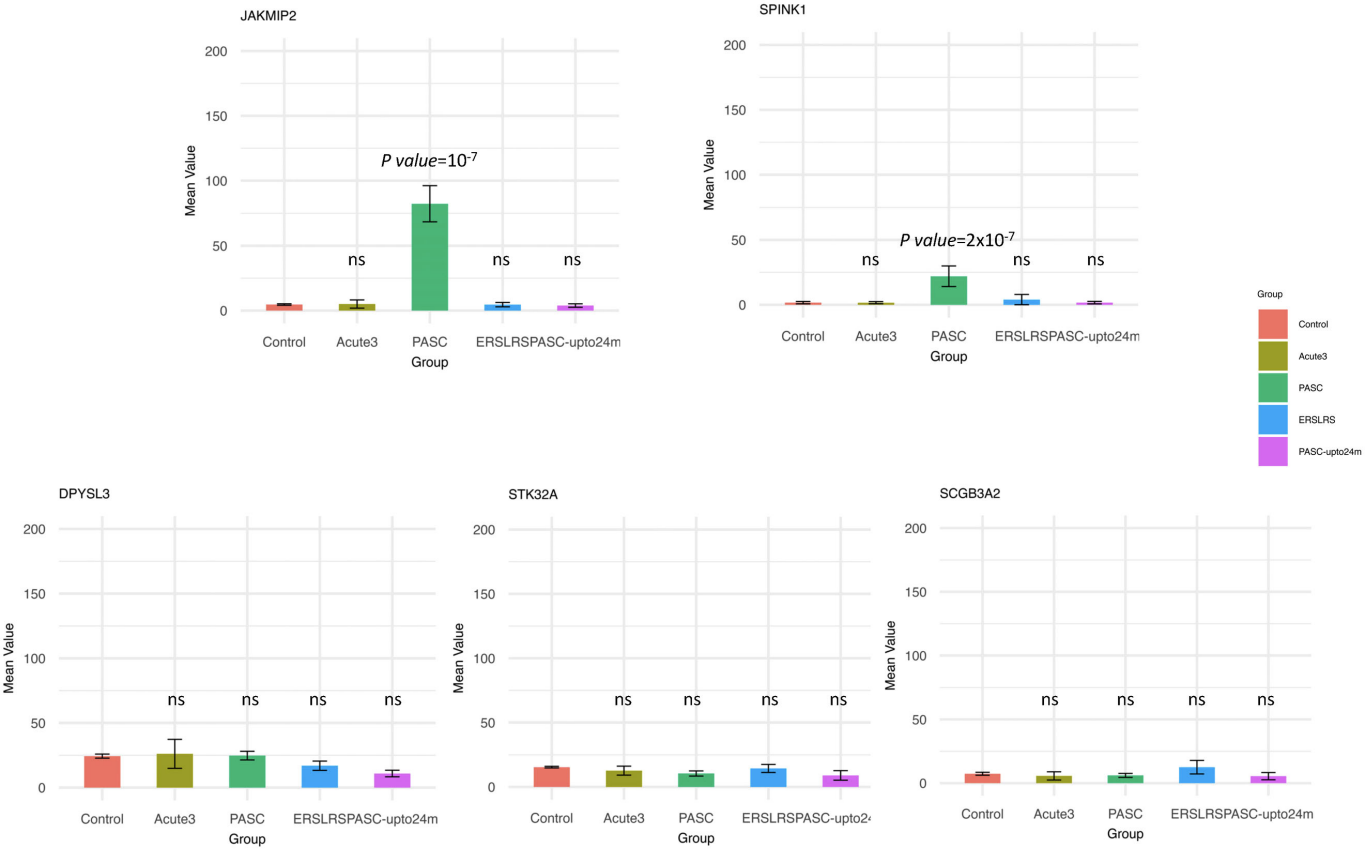
Analysis of host gene expression in control, acute, early/late, PASC 24 month and 8-month PASC. For each host gene, the sequence depth observed in each patient group was compared to the Control group using ANOVA followed by Tukey’s HSD test. Any statistically significant p-value (p value < 0.05) are shown in the figure (‘ns’ indicates not significant). Two genes (SPINK1 and JAKMIP2) exhibited significantly higher read depth in the PASC group.

Since the HERV-001 locus was the most amplified, we next compared the expression of JAKMIP2 and the neighboring genes: STK32A, DPYSL3, SPINK1, and SCGB3A2. Using ANOVA followed by Tukey’s HSD test, we compared the following groups: control, acute3, PASC, ERS-LRS, and PASC-upto24m (**Figure 9**). From this analysis, we identified 3 genes (DPYSL3, JAKMIP2, and SPINK1) with significantly higher good/usable window numbers in PASC group. Additionally, 2 genes (SPINK1 and JAKMIP2) exhibited significantly higher read depth in the PASC group (**Figure 10**). In contrast, the host genes at a greater distance from JAKMIP2 and SPINK1, the expression as determined by number of windows and read depth of STK32A, DPYSL3 and SCGB3A2 were not different between control, acute 3 PASC, ERS-LRS and PASC-upto24m (**Figure 9**, **Supplementary Table 9**). Collectively, these results establish a pattern of co-amplified transcription of select HERV loci and neighboring host genes, consistent with localized epigenetic remodeling known to occur following COVID-19 infection (20, 49).

## Discussion

In this study, we used our previously described WHA to characterize the expression of HERV loci in monocytes from healthy individuals and patients with infections, including COVID-19, influenza, sepsis and Dengue virus. We identified HERV loci that were expressed in infected patients compared to normal individuals and those who were exposed but did not develop an infection. We also analyzed post COVID-19 infection and found extensive positive HERV loci in monocytes from patients after infection and patients with PASC 8 months after acute COVID-19. Distinct patterns of HERV loci expression, determined by the number of good/usable windows and sequence depth, were observed when comparing acute COVID-19 and PASC patients. Finally, in these PASC patients, we demonstrate that the presence of localized transcription where both host genes and HERV loci were co-amplified due to localized epigenetic modification known to occur because of the expansion of myeloid precursors following COVID-19 infection.

In a previous study, we analyzed a scRNA-seq dataset taken from PBMC that had been stimulated *in vitro* with LPS, a known activator of innate immune cells such as monocytes (50–52). Using the WHA, we identified numerous HERV loci that were positive only in the monocytes from LPS-stimulated PBMC, but not in resting PBMC (30). We then used the WHA with scRNA seq PBMC datasets from a panel of healthy individuals to establish a pangenome control set with negative HERV loci (30). Comparing patients with acute COVID-19 or trauma to this pangenome control revealed positive HERV loci expression in monocytes from individuals with acute COVID-19 and those who had experienced individuals that had physical trauma. In the current study, we expanded our WHA analysis to include two additional scRNA-seq datasets from individuals with infections caused by acute COVID-19, influenza, Dengue virus, and sepsis. For each disease, we identified HERV loci that were positive in patients but negative in the 31-pangenome controls. We observed differences in the numbers of positive HERV loci from examination of individuals with the different diseases. A possible explanation for this variation could be the timing of sample collection. Given the short half-life of circulating monocytes, the interaction between the pathogen and the immune system at different time points could influence the extent of innate immune activation (53). Although we found considerable variation in the number of positive HERV across samples, the highest numbers were observed in patients with respiratory viral infections, specifically COVID-19 and influenza. Previous studies have demonstrated that respiratory viral infections are particularly effective at stimulating the innate immune system (5, 54, 55). Even the COVID-19 and influenza vaccines led to the expression of positive HERV loci, suggesting that these viral proteins in the absence of active respiratory infection can activate innate immunity, although the effect may also be influenced by the adjuvants co-administered with these vaccines that can also stimulate innate immunity (14).

One of the most striking findings in our study was the identification of positive HERV loci in patients even after the resolution of the acute phase of COVID-19. A sporadic pattern of HERV loci expression was observed in samples from patients at both early and late time points following acute infection as determined by RT-PCR analysis. The timing of sample collection after the acute infection in these patients then was well beyond the half-life of circulating monocytes (53). Even more remarkable, positive HERV were detected in all patients from a scRNA-seq analysis of samples taken 8 months post-acute COVID-19, with 3 specific HERV loci positive across all 12 patients that were a subset of a larger cohort (46). Yin et al. reported that 8 of these 12 patients exhibited symptoms consistent with long-term COVID-19 at month 8, while the remaining 4 had post-COVID-19 symptoms that had resolved by that time (41, 42, 46). Although no specific patient characteristics explained the clustering of these 12 individuals, the study’s inclusion criteria may have biased the sample toward patients with unresolved symptoms (41, 42). In other words, patients experiencing persistent symptoms were more likely to remain in the study to gain insights into resolving their post COVID-19 symptoms (41, 42). Support for the uniqueness of the 12 PASC samples comes from the analysis of a second scRNA-seq dataset with patients at 4, 8, and 24 months post-COVID-19 (47). In this study, different criteria were used to define long-term COVID-19, and normal patient samples were combined with experimental samples for scRNA-seq analysis. Despite these methodological differences, a few positive HERV loci were detected at the 4- and 8-month time points, but none were observed at 24 months.

Further analysis of the 12 individuals with PASC revealed that some of the positive HERV loci in the 12 PASC samples had a greater number of good/usable windows (indicative of longer transcript lengths), and sequence read depth when compared to samples from patients with acute COVID-19. One that stands out is the good window/depth amplification found in HERV001 that is located between introns 1–2 of the JAKMIP2 gene. In acutely infected COVID-19 patients, we found parallel expression of JAMMIP2 and HERV001 in a few samples, while in the PASC patients we found amplification of both JAKMIP2 and HERV001. Consistent with these results, a previous study noted that JAKMIP2 expression occurs in a temporal fashion in pluripotent stem cells during development (56). From a region analysis of gene expression surrounding JAKMIP2-HERV001, we also found significant expression of the gene serine peptidase inhibitor Kazal type 1 (SPINK1) in the monocytes from PASC patients. A previous study demonstrated that silencing of SPINK1 suppressed the proliferation of hepatocellular carcinoma cells leading to the possibility that SPINK1 possessed oncogenic properties (57). Interestingly, we found that the expression of several other genes at a further distance from the JAKMIP2-HERV001-SPINK1 genes did not show significant increase in expression in the PASC patients from the controls. Since previous studies have shown viral lung infections can result in the epigenetic modification of myeloid precursors following COVID-19 infection that could last for up to a year after infection, our analysis demonstrating the localized epigenetic remodeling and re-expression of previously silenced JAKMIP2-HERV001-STINK1 might have a profound effect on monocyte function (10, 19, 58–62). Further studies will be required to assess the extent of host cell expression in the same chromosome locale of additional expressed HERV loci and the impact of that the expression of host genes might have on the patients with PASC.

## Conclusion

Our results characterize the expression of select HERV loci following viral infections, which were not expressed in normal monocytes from patient controls. We found distinct patterns of expressed HERV loci in monocytes from PBMC across 3 acute COVID-19 datasets. These patterns were also different from those found in patients with influenza infections, Dengue virus infections and sepsis and in individuals who received the COVID-19 or influenza vaccine. Collectively, these findings are consistent with our previous study and support a patient specific expression pattern for the HERV loci (30). However, in contrast to the acute COVID-19 infections, the PASC datasets revealed several expressed HERV loci that were shared across multiple patients. We acknowledge limitations in our analysis. First, we used a limited number of studies because our analysis relied on publicly available scRNA-seq datasets where raw sequencing data were also accessible. Second, the COVID-19 datasets included patient sample sets that were not standardized across different studies. Importantly, for the analysis of post COVID-19 patients, Peluso et al. pointed out that substantial variability exists in the clinical features of PASC, suggesting that sub-phenotypes may exist, which could impact comparisons between different studies (41, 42). Finally, although the 31-control sample set contained a range of ages that overlapped in general with the patient samples, Mao et al. has recently demonstrated the contribution of age and epigenetic modifications to the reactivation of certain HERV (31). To overcome these limitations, future analyses with longitudinal sampling will be essential to track the patterns of select HERV loci expression and correlate them with specific features of PASC. Even with these limitations, our studies establish patterns of co-amplified HERV loci and neighboring host gene transcripts in monocytes from patients with PASC, providing a new prospective on understanding the clinical features of long term COVID-19. Further delineation of the extent of these HERV-host gene patterns in PASC, which are most likely due to the localized epigenetic remodeling of myeloid progenitor cells, will be necessary to better correlate these findings with the clinical features of long-term recovery from COVID-19.

## Materials and methods

### Datasets used in this study

In this study, we used 14 publicly available scRNA-seq datasets from 1) 6 healthy individuals from Amrute et al. (13), 2) 2 healthy individuals from Derbois et al. (50), 3) 10 healthy individuals from Chen et al. (63), 4) 3 healthy individuals from Thompson et al. (64), 5) 4 healthy individuals from Lee et al. (35) and 6) 5 healthy individuals from Yu et al. (34). For patients’ data, we obtained publicly available scRNA-seq datasets from 1) 11 COVID-19 patients and 5 patients infected with influenza A virus from Lee et al. (35), 2) 10 COVID-19 patients from Unterman et al. (7), 3) 12 hospitalized COVID-19 patients, with samples collected at both day 0 and day 7 of enrollment from Amrute et al. (13), 4) 3 individuals who were in close contact with COVID-19 patients (exposed) and 10 outpatients (infected) who confirmed COVID-19 from Yu et al. (34), 5) 6 individuals who received two doses of an mRNA COVID-19 vaccine without prior SARS-CoV-2 infection from Terzoli et al. (39), 6) 1 individual who received an influenza vaccine without having been vaccinated against influenza for at least 3 years from Turner et al. (65), 7) Early Recovery Stage (ERS) and Late Recovery Stage (LRS) COVID-19 patients from Wen et al. (40), 8) 12 PASC patients who consistently met the case definition for LC symptoms for 8 months following COVID-19 infection from Yin et al. (46), 9) 2 COVID-19 patients followed for 24-months from diagnosis, with sample collected at up to 8 pre-specified time points from Phetsouphanh et al. (47), 10) 4 sepsis patients and 3 sepsis patients with Acute Respiratory Distress Syndrome (ARDS) from Jiang et al. (6), and 11) 15 dengue patients from Ghita et al. (38) Detailed sequence reads information for each sample is listed in **Supplementary Table 10**.

### scRNA-seq data with WHA analysis

The scRNA-seq PBMC datasets were downloaded in either fastq or bam format, depending on availability from NCBI SRA or GEO. If only bam files were available, they were converted to fastq format using 10x Genomics Cell Ranger software (v 7.1.0) bamtofastq (v 1.4.1) (66). All fastq files were aligned to the human reference genome (GRCh38) using Cell Ranger count with default parameter settings. Monocytes were then selected based on matrix files generated by Azimuth (https://satijalab.org/azimuth/) (37), which normalizes gene expression data and performs clustering to predict cell identity. To extract sequence reads corresponding to monocytes, barcodes associated with these cells were listed in.txt file and used to filter bam files with samtools (v 0.1.19). The resulting.bam files were then converted to fastq files using bedtools (v 2.26.0).

Processed fastq files were then used for Window-based HERV Alignment (WHA) analysis. Each sample was duplicated for WHA analysis and mapped to 3,220 HERV loci as reference data (**Supplementary Table 11**) using BWA (v 0.7.13) with minimum percent match threshold of >99% (30). First, we selected HERV loci with 100% WSS scores. From these, loci with a sequence depth of less than 3 or fewer than 9 usable windows were classified as negative HERV loci. Conversely, loci with a sequence read depth greater than 3 and at least 9 usable windows were classified as positive HERV loci.

The HERV profiles of healthy individuals and patients were compared through manual filtering using Excel files to identify HERV loci unique to patient samples, ensuring that all control samples excluded any positive HERV loci. To achieve this, we selected only negative values for all healthy individuals, while ensuring that at least one positive value remained in the patient samples. From this analysis, a total of 208 HERV loci were selected for further analysis. Heatmaps were generated to visualize differences in HERV loci across various groups using STAMP (67), and R (68).

To determine significant differences in sequencing depth and good/usable windows between the acute COVID-19 group and the long-term COVID-19 group, we performed a t-test using the ggplot2 package in R (https://cran.r-project.org/web/packages/ggpubr/index.html). A P value < 0.05 was considered statistically significant for this analysis.

### Host gene analysis

To further investigate the genes, STK32A, DPYSL3, JAKMIP2, SPINK1, and SCGB3A2, which are located near our HERV locus of interest (HERV-001), we reprocessed the fastq files (monocytes PBMC) used in our WHA analysis. This time, all files were mapped using each gene as reference data. For each group (control, acute3, PASC, ERS/LRS, and PASC-upto24m), we recorded the sequence depth and number of good/usable windows for each gene. Additionally, we performed an ANOVA statistical analysis followed by Tukey’s HSD test to determine statistical significance (P value < 0.05) for each gene.

## Data Availability

The original sequencing dataset used in this study were downloaded from NCBI GEO or NGDC. Accession numbers are as follows: GSE192391, GSE226488, GSE162806, GSE166992, GSE149689, GSE171555, GSE155224, GSE260763, GSE148633, PRJCA002413, GSE235050, GSE262861, GSE151263, GSE220969.

## Supplementary material

Supplementary Figure 1 Comparison of the number of positive windows obtained from PASC patients and those found in the remaining datasets. The average number of good/usable windows, along with the standard deviation, observed in the PASC samples is shown in the figure.

Supplementary Figure 2 Comparison of the number of positive windows obtained from PASC patients and those found in the remaining datasets. **(A)** The average number of good/usable windows, along with the standard deviation, observed in the PASC samples is shown in the figure. **(B)** Positive HERV loci identified not in the 8-month PASC group, but only in the remaining groups.

Supplementary Figure 3 Comparison of the sequence read depths of PASC with the remaining data sets. Comparison of the read depths for two HERV loci between PASC and the remaining Acute COVID group using t-test.

Supplementary Figure 4 Chromosomal distribution of HERV loci. This figure shows the full chromosomal map illustrating the distribution of all identified HERV loci across the human genome. The specific regions highlighted in red/blue correspond to the HERV loci used in **Figures 6A and B**. All other black markers represent the full set of HERV loci analyzed in this study. See **Supplementary Table 11** for the full set of HERV genome coordinates.
