# Supplementary material for: Amplification of select autonomous HERV loci and surrounding host gene transcription in monocytes from patients with post-acute sequelae of COVID-19
Source: Front Immunol. 2025 Jun 26;16:1621657. doi: 10.3389/fimmu.2025.1621657 (PMC12241865; doi:10.3389/fimmu.2025.1621657)
Supplement: Supplementary Figure 1 — Comparison of the number of positive windows obtained from PASC patients and those found in the remaining datasets. The average number of good/usable windows, along with the standard deviation, observed in the PASC samples is shown in the figure. [file DataSheet1.pdf]

Amplification of select autonomous HERV loci and surrounding host gene transcription in monocytes from patients with post-acute sequelae of COVID-19

Hyunmin Koo and Casey D. Morrow

Supplementary Method

In a previous study, we have described the WHA tool [1]. The WHA tool, which was modified from our previous tool, called WSS. For the WSS analysis, sequence reads from each sample were aligned to the 93 microbial reference genomes which were previously established based on the HMP dataset [2, 3] using the Burrows-Wheeler aligner (BWA) tool [4]. Each sample was analyzed for multi-sample SNVs relative to the provided reference genome using the Genome Analysis Toolkit (GATK) [5]. The resulting multi-sample Variant Call Format (VCF) files were utilized for pairwise comparisons between all possible pairs of samples. This was done to determine the overall genome-wide SNV similarity for each microbial species. Samples with sequence coverage below 30% and sequence depth less than 3.5 against their given reference genome were excluded from the pairwise comparisons [2, 6-12]. In order to identify related strains, the WSS score was compared to a previously established cut-off value from our earlier study. (For related strain pairs: WSS score>cut-off; for unrelated strain pairs: WSS score<cut-off) [2, 13].

In WSS, a window is defined as similar if the SNV pattern is exactly the same between compared two samples or no SNV is present in both samples. Good (usable) windows are defined when each window had more than 50% of the bases having a satisfied coverage and depth value compared to cut-off values.

Window-based HERV Alignment (WHA) analysis.

Compared to the WSS, we replace the reference genomes from microbes with 3,200 HERV loci. We also added new codes that can cluster cell-type specific hashtags and then filter the selected cell-type related sequence reads for pre-processing. Then, the same alignment tool was used to map HERV reference loci with different minimum percent matches, 99%. Each window size was adjusted from 100 base pairs to 50 base pairs, sequence depth cut-off was

adjusted from 5 to 3, and a new cut-off value (total good/usable windows >8 representing at least 400 base pairs of DNA sequence) to identify positive HERV loci. For the analysis presented in "**Amplification of select autonomous HERV loci and surrounding host gene transcription in monocytes from patients with post-acute sequelae of COVID-19" we did the following steps (Note further details can be found in Supplementary Figure 1 of [1]):**

1. We used a publicly available scRNA-seq datasets from previously published studies.
2. Raw DNA sequencing files were downloaded and analyzed using the 10X Genomics pipeline along with Seurat, applying the Azimuth reference to identify monocyte-associated transcriptomic profiles.
3. Sequence reads were aligned to 3,200 known HERV loci, as previously described in Koo and Morrow [1] and other studies.
4. Window-based HERV Analysis (WHA) was performed for each sample to evaluate read depth and usable window counts at each HERV locus.
5. Those samples with read depth below 3 and windows below 8 are designated as negative, where those with read depth 9 or above are with read depth greater than 3 are designated as positive. This thresholding approach is also explained in the first paragraph of the Results and in the Methods.
6. To control for background signal, all HERV loci detected in any of the 31 healthy control samples (from our previous study) were excluded from downstream disease comparisons (even one plus in the 31 samples).
7. We report any positive HERV identified in the individual with the disease dataset that is not positive in even one of the individuals in the control dataset.

Note that in our previous publication, the dataset from the 31 individuals contained over 1 billion sequence reads used to identify HERV loci expressed in the control samples [1]. We also noted that the differences between HERV loci transcriptome expression patterns in the

disease samples from patients versus control samples did not correlate with the DNA sequence read count. See Supplementary Figure 3 in [1].

References

1. Koo, H. and C.D. Morrow, *Shared and unique patterns of autonomous human endogenous retrovirus loci transcriptomes in CD14+ monocytes from individuals with physical trauma or infection with COVID-19.* Retrovirology, 2024. **21**(1): p. 17.
2. Kumar, R., et al., *Identification of donor microbe species that colonize and persist long term in the recipient after fecal transplant for recurrent Clostridium difficile.* NPJ biofilms and microbiomes, 2017. **3**(1): p. 12.
3. Schloissnig, S., et al., *Genomic variation landscape of the human gut microbiome.* Nature, 2013. **493**(7430): p. 45-50.
4. Li, H. and R. Durbin, *Fast and accurate long-read alignment with Burrows–Wheeler transform.* Bioinformatics, 2010. **26**(5): p. 589-595.
5. Van der Auwera, G.A., et al., *From FastQ data to high confidence variant calls: the Genome Analysis Toolkit best practices pipeline.* Curr Protoc Bioinformatics, 2013. **43**: p. 11.10.1-33.
6. Koo, H., D.K. Crossman, and C.D. Morrow, *Strain Tracking to Identify Individualized Patterns of Microbial Strain Stability in the Developing Infant Gut Ecosystem.* Frontiers in Pediatrics, 2020. **8**.
7. Koo, H., et al., *Individualized recovery of gut microbial strains post antibiotics.* NPJ Biofilms Microbiomes, 2019. **5**: p. 30.
8. Koo, H., et al., *Sharing of gut microbial strains between selected individual sets of twins cohabitating for decades.* PLOS One, 2019. **14**(12): p. e0226111.
9. Koo, H., et al., *An individualized mosaic of maternal microbial strains is transmitted to the infant gut microbial community.* Royal Society Open Science, 2020. **7**: p. 192200.

10. Koo, H. and C.D. Morrow, *Perturbation of the human gastrointestinal tract microbial ecosystem by oral drugs to treat chronic disease results in a spectrum of individual specific patterns of extinction and persistence of dominant microbial strains.* PLOS One, 2020. **15**(12): p. e0242021.

11. Koo, H. and C.D. Morrow, *Bacteroidales-specific antimicrobial gene analysis identifies gastrointestinal tract reservoirs of microbial sub strains selected for fecal dominance.* PREPRINT (Version 1) available at Research Square, 2022.

12. Koo, H. and C.D. Morrow, *Time series strain tracking analysis post fecal transplantation identifies individual specific patterns of fecal dominant donor, recipient, and unrelated microbial strains.* Plos one, 2022. **17**(9): p. e0274633.

13. Kumar, R., et al., *New microbe genomic variants in patients fecal community following surgical disruption of the upper human gastrointestinal tract.* Human Microbiome Journal, 2018. **10**: p. 37-42.
