# Supplementary material for: Amplification of select autonomous HERV loci and surrounding host gene transcription in monocytes from patients with post-acute sequelae of COVID-19
Source: Front Immunol. 2025 Jun 26;16:1621657. doi: 10.3389/fimmu.2025.1621657 (PMC12241865; doi:10.3389/fimmu.2025.1621657)
Supplement: Supplementary file 2 [file Presentation2.pptx]

## Slide 1
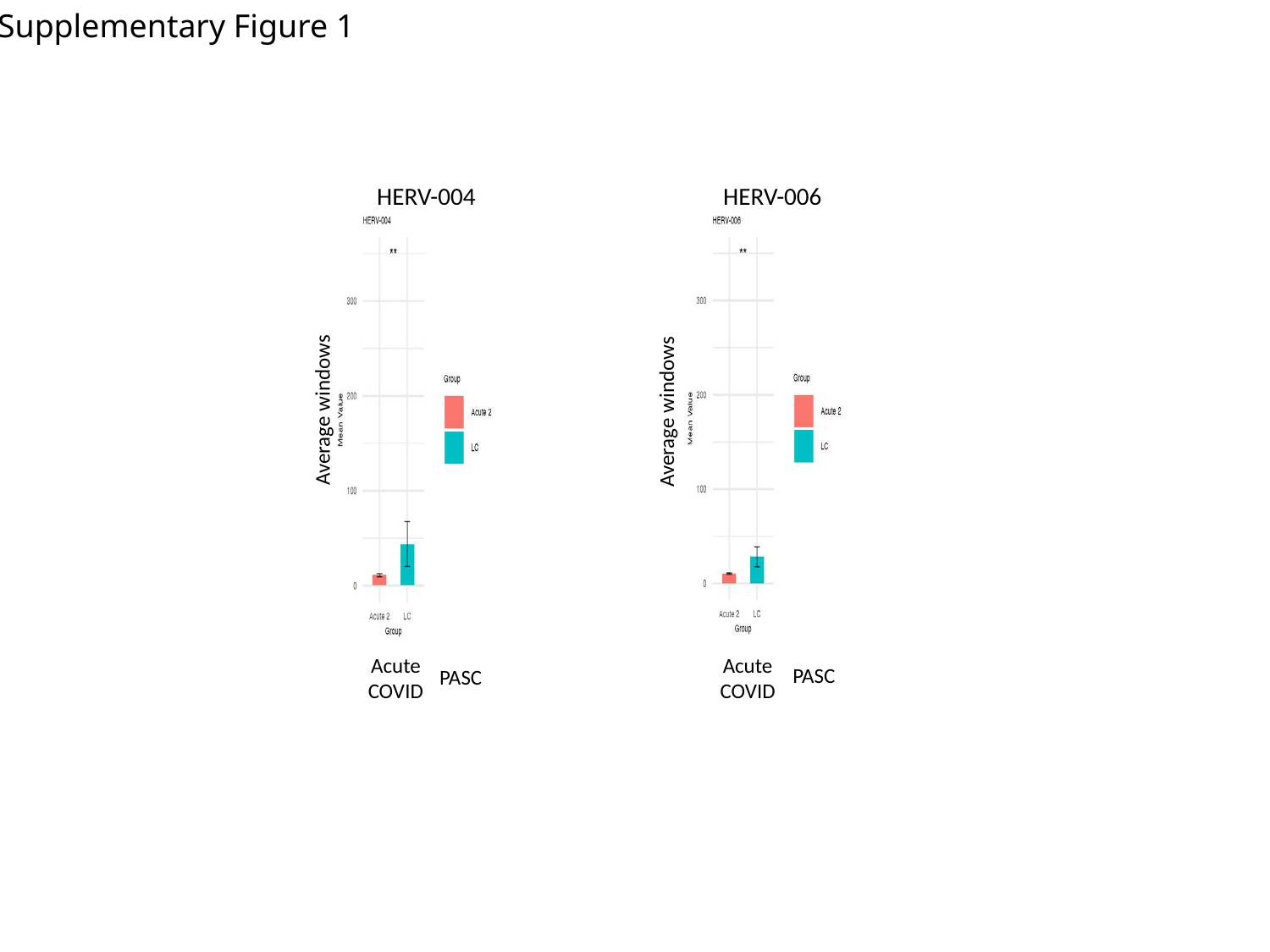

Supplementary Figure 1
HERV-004
Average windows
Acute
COVID
PASC
HERV-006
Average windows
Acute
COVID
PASC

## Slide 2
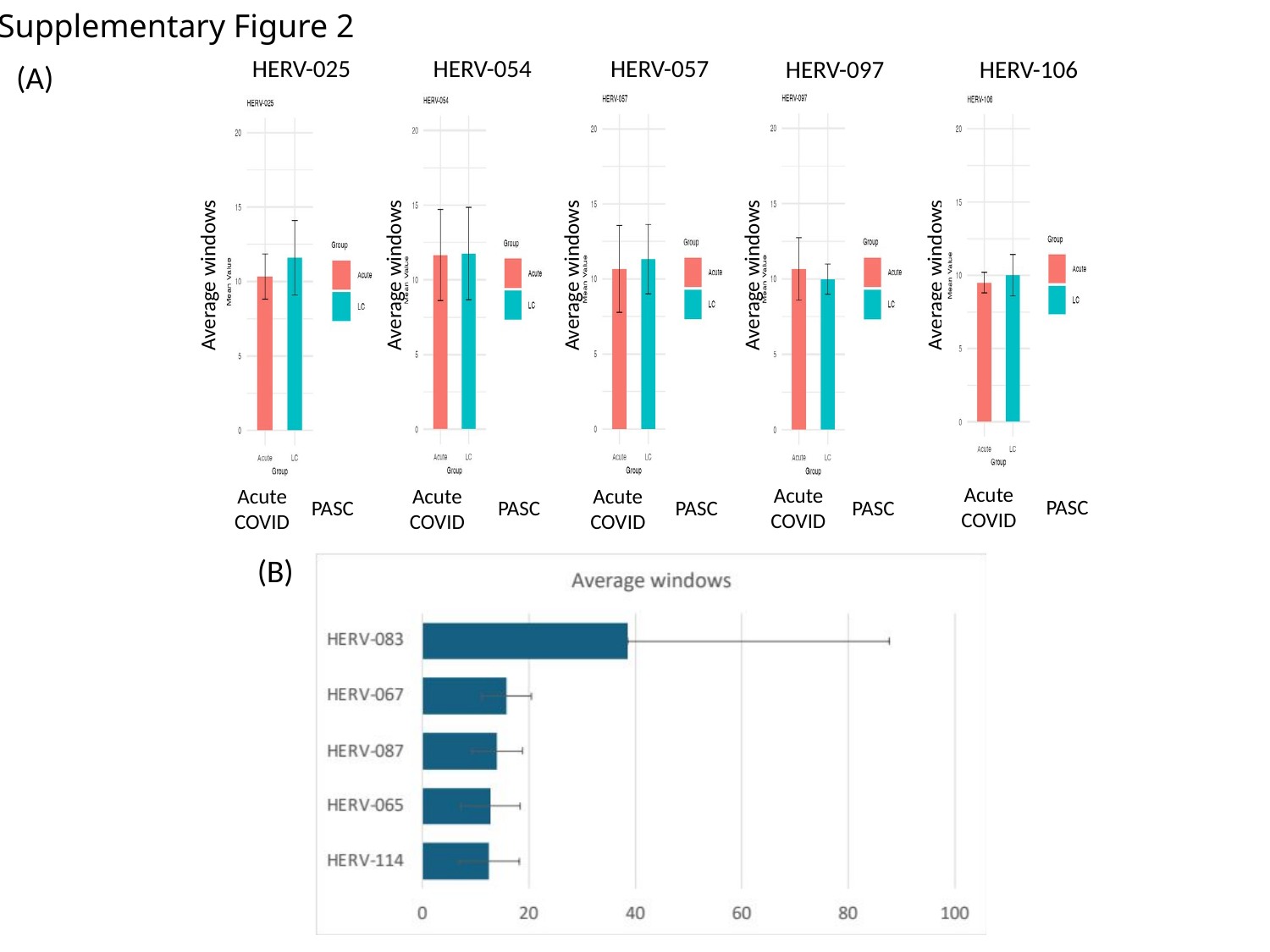

Supplementary Figure 2
HERV-025
HERV-054
HERV-057
HERV-097
HERV-106
(A)
Average windows
Average windows
Average windows
Average windows
Average windows
Acute
COVID
Acute
COVID
Acute
COVID
Acute
COVID
Acute
COVID
PASC
PASC
PASC
PASC
PASC
(B)

## Slide 3
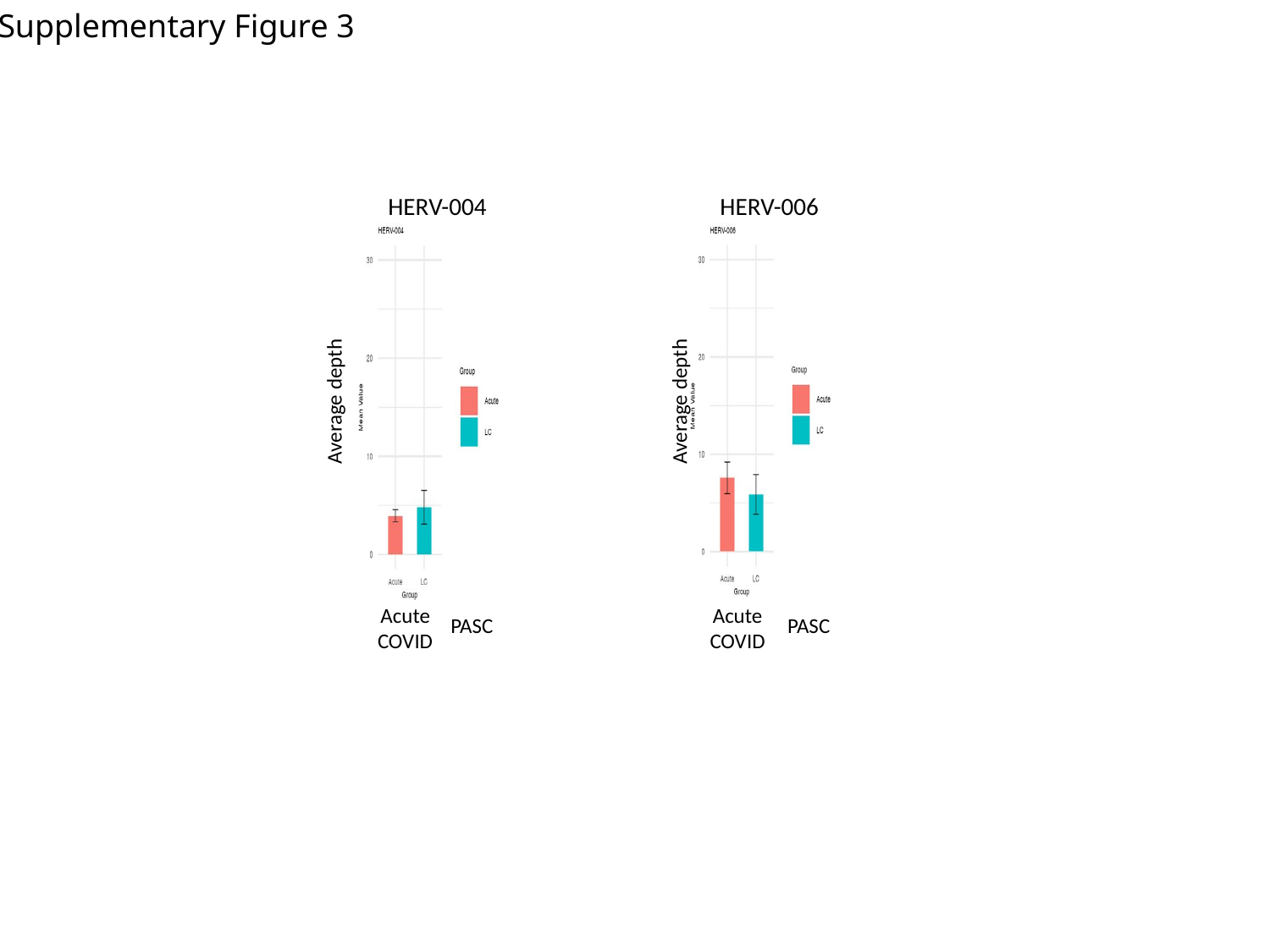

Supplementary Figure 3
HERV-004
Average depth
Acute
COVID
PASC
HERV-006
Average depth
Acute
COVID
PASC

## Slide 4
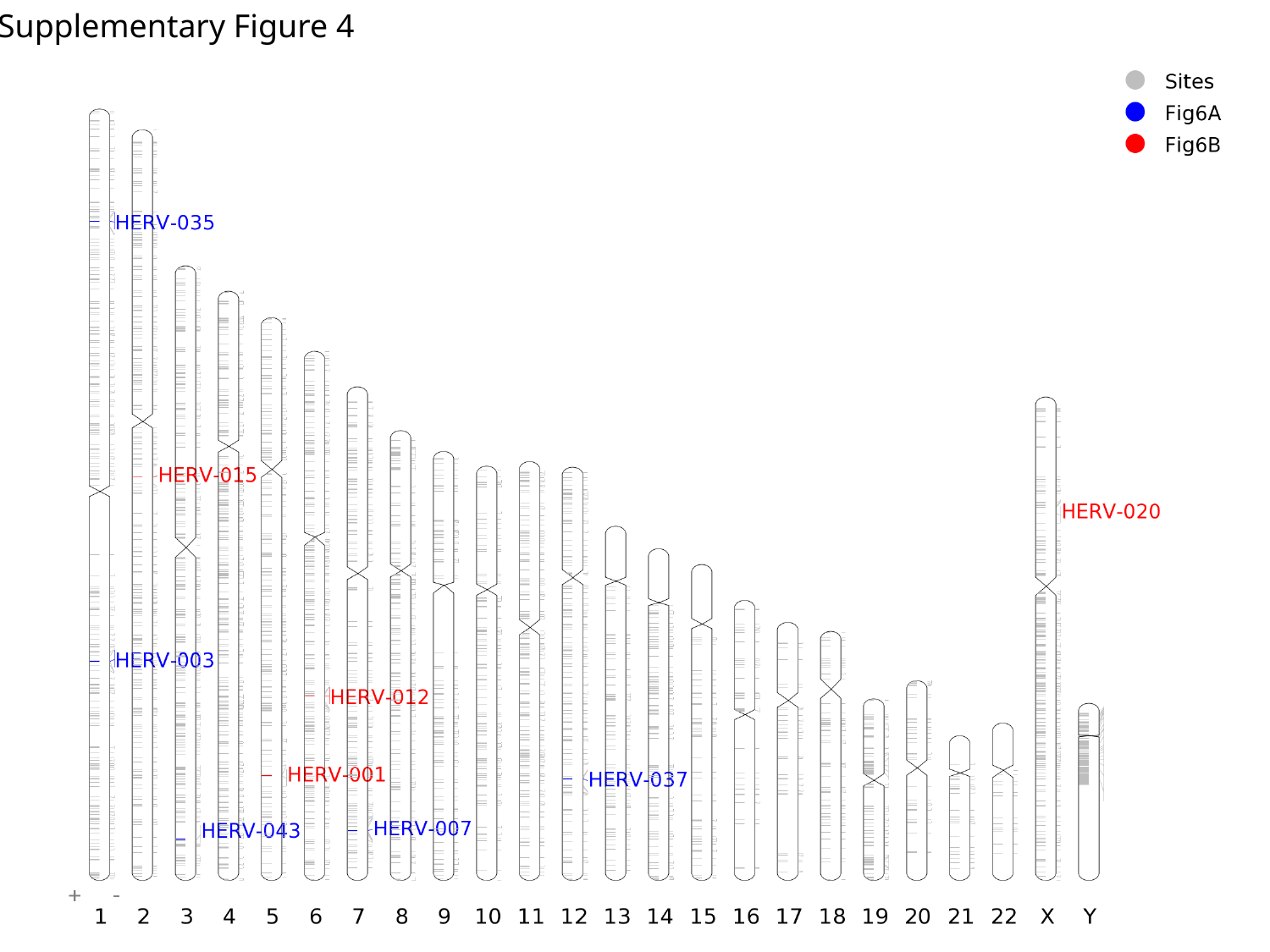

Supplementary Figure 4
